# Label-free, automated classification of microsatellite status in colorectal cancer by infrared imaging

**DOI:** 10.1038/s41598-020-67052-z

**Published:** 2020-06-23

**Authors:** Angela Kallenbach-Thieltges, Frederik Großerueschkamp, Hendrik Jütte, Claus Kuepper, Anke Reinacher-Schick, Andrea Tannapfel, Klaus Gerwert

**Affiliations:** 1 Ruhr University Bochum, Center for Protein Diagnostics (ProDi), Biospectroscopy, Bochum, Germany; 2 Ruhr University Bochum, Faculty of Biology and Biotechnology, Department of Biophysics, Bochum, Germany; 3 Institute of Pathology, Ruhr University Bochum, Bochum, Germany; 4 Department of Hematology, Oncology and Palliative Care, St. Josef Hospital, Ruhr University Bochum, Bochum, Germany

**Keywords:** Cancer imaging, Colorectal cancer, Cancer imaging, Colorectal cancer

## Abstract

Challenging histopathological diagnostics in cancer include microsatellite instability-high (MSI-H) colorectal cancer (CRC), which occurs in 15% of early-stage CRC and is caused by a deficiency in the mismatch repair system. The diagnosis of MSI-H cannot be reliably achieved by visual inspection of a hematoxylin and eosin stained thin section alone, but additionally requires subsequent molecular analysis. Time- and sample-intensive immunohistochemistry with subsequent fragment length analysis is used. The aim of the presented feasibility study is to test the ability of quantum cascade laser (QCL)-based infrared (IR) imaging as an alternative diagnostic tool for MSI-H in CRC. We analyzed samples from 100 patients with sporadic CRC UICC stage II and III. Forty samples were used to develop the random forest classifier and 60 samples to verify the results on an independent blinded dataset. Specifically, 100% sensitivity and 93% specificity were achieved based on the independent 30 MSI-H- and 30 microsatellite stable (MSS)-patient validation cohort. This showed that QCL-based IR imaging is able to distinguish between MSI-H and MSS for sporadic CRC - a question that goes beyond morphological features - based on the use of spatially resolved infrared spectra used as biomolecular fingerprints.

## Introduction

Colorectal cancer (CRC) is the third most prevalent cancer and a leading cause of cancer-related deaths worldwide^1,2^. In recent years, improvements in treatment have been based on the understanding of molecular heterogeneity at multiple levels including genomics, epigenomics, and transcriptomics. Especially, understanding features of the microenvironment will ultimately improve treatment^3^. Colorectal cancer can be classified as either chromosomally instable or microsatellite instable (MSI). Notably, 15% of all UICC stage II and III CRCs are high MSI (MSI-H) showing hypermutated tumors. This results from a defective DNA mismatch repair (MMR) system, having a clear molecular origin (MLH1, MSH2, MSH6, and PMS2 inactivation), and arises from a germline or somatic mutations^4–6^.

MSI-H is a strong prognostic factor in early colon cancer, and especially stage II disease, but its power is diminished in stage III^7–10^. The microenvironment of MSI-H cancers is densely infiltrated by CD8-positive cytotoxic T-lymphocytes and activated T-helper 1 (Th1) cells through presentation of multiple neoantigens by hypermutated tumor cells. Therefore, MSI-H cancers are particularly sensitive to immune checkpoint inhibitors such as pembrolizumab^11,12^. This drug was approved by the FDA for the treatment of advanced MSI-H CRC in 2018. Therefore, a fast and reliable screening method for MSI-H would have a marked impact on precise treatment.

In recent years, new approaches for MSI-H detection have emerged using next generation sequencing (NGS) datasets with different strategies such as read-count distribution (97.9% sensitivity and 100% specificity compared to those with fragment length analysis)^13^, computing the length distributions of microsatellites per site in paired tumor and normal sequence data^14^, or by comparing the number of repeats in different microsatellite loci (use of three different NGS panels resulting in a sensitivity range of 96.4% to 100% and a specificity range of 97.2% to 100% compared to those with fragment length analysis)^15^. NGS for MSI-H has not yet been used in routine clinical diagnostic procedure owing to it being costly and laborious. Recently, digital pathology was presented to classify MSI-H status using two hierarchically applied deep convolutional neural networks on hematoxylin and eosin (H&E)-stained images, reaching a patient-level area under the curve (AUC) of 0.84. The reference for this study was fragment length analysis^16^.

The microsatellite status of CRC is routinely determined by loss of expression of one or more MMR proteins, which can be visualized using immunohistochemistry (IHC)^17^. The technical performance of MMR IHC can vary widely depending on fixation, antigen retrieval, primary antibody, and staining platform^18^. Therefore, subsequent fragment length analysis could be used to validate the diagnosis of low-level MSI (MSI-L) and MSI-H. Fragment length changes are determined for mononucleotide markers BAT25 and BAT26, as well as dinucleotide markers D2S123, D5S346, and D17S250. Thereafter, cases with instability in two or more genes are classified as MSI-H, whereas those with instability in a single gene are classified as MSI-L, according to the current Bethesda criteria^19,20^. This diagnostic workflow is sample- and time-consuming. Specifically, it takes up to five thin tissue sections and several hours to obtain a clear-cut diagnosis. To streamline patients for this sophisticated approach, we applied label-free digital pathology as a novel initial screening method to identify MSI-H. This automated approach does not consume tissue material, has no inter-/intra-observer variability, and can additionally classify the tissue microenvironment within 30 min.

Classification using infrared (IR) imaging has been reported for several types of tissue such as lung (including its subtyping)^21^, liver^22^, brain^23^, bladder^24,25^, prostate^26^, breast^27^, and colon^28,29^, along with its grading^30,31^. To date, IR imaging employs the measurement of spatially-resolved infrared spectra as fingerprints of integral biochemical cellular composition to represent morphological changes^32–34^. Here, spectra are automatically classified using an established bioinformatics workflow^30^.

Conventional Fourier transform infrared (FTIR)-based microscopes were used for most of these studies. Recently, we and other groups have shown that quantum cascade laser (QCL)-based IR imaging is much faster compared to FTIR systems^35–39^. It was demonstrated that thin tissue sections can now be classified within a few minutes instead of several hours using FTIR-based imaging^40,41^. An in-depth quantitative comparison of FTIR- and QCL-based imaging was performed by Bhargava *et al*.^42^ Unfortunately, coherence effects disturb QCL-based IR images; thus spectral quality is lower than that in FTIR-based images. Coherence effects in imaging have been reported in literature^43^. In our previous study, QCL-based IR imaging and FTIR-based imaging were compared for use with CRC tissue sections. We used a commercially available QCL-based IR microscope for fast label-free CRC tissue imaging to identify cancer in whole slide sections reaching 96% sensitivity and 100% specificity^44^. With this, cancerous regions could be distinguished from other pathological regions such as inflammation and healthy regions representing morphological features. However, a more challenging diagnosis based on alterations at the molecular level has not been presented for IR imaging on CRC. The open question that we addressed here is whether QCL-based IR imaging is suitable to provide a challenging diagnosis based on molecular changes only. To answer this, we provided a classification based on a diagnostic issue, wherein H&E-stained images indicating morphological alterations are not sufficient for diagnosis, and in addition, molecular information is needed.

In this feasibility study, QCL-based IR imaging was used to classify the microsatellite status for CRC. The correlation between FTIR and molecular data has been reported previously, through stain-less staining for protein biomarkers^45^. Here, we show that IR imaging can resolve molecular changes associated with disease due to defective DNA mismatch repair in the cells that are not accessible by morphological evaluation. These subtle changes are resolved currently by IHC with subsequent fragment length analysis for MSI-H. Regarding performance, the QCL-based IR microscope, as compared to these advanced established approaches in pathology, is much faster and much less sample-consuming. To this end, we analyzed thin tissue sections of UICC stage II and III CRC tumors from 100 patients (Table 1). The tumor status of all samples was known. The microsatellite status for 40 tumor patients was also known (19 MSI-H and 21 MSS). The remaining 60 tumor samples (after analysis unblinded as 30 MSI-H and 30 MSS) were used as a blinded verification set for the MSI-H/MSS classification. The label-free digital pathology approach achieved 100% sensitivity and 93% specificity as compared to IHC with subsequent fragment length analysis (FLA).

**Table 1:** The table shows the IHC, PCR and IR imaging results for the 60 independent validation patients.

Patient	MLH1%	MSH2%	MSH6%	PMS2%	MSI-FLA	BAT25	BAT26	D17S250	D2S123	D5S346	IR imaging	IR MSI-H positive %
V01	80	90	85	80	IHC +						MSS	62,38
V02	20	90	60	50	IHC +						MSS	39,87
V03	90	85	95	85	IHC +						MSS	52,30
V04	80	85	70	65	IHC +						MSS	60,78
V05	80	70	50	60	IHC +						MSS	43,35
V06	95	95	60	75	IHC +						MSS	56,04
V07	95	95	70	85	IHC +						MSS	58,90
V08	90	90	90	90	IHC +						MSS	59,04
V09	90	90	50	70	IHC +						MSS	54,99
V10	95	90	60	85	IHC +						MSS	46,81
V11	95	95	90	80	IHC +						MSS	47,30
V12	90	90	60	80	IHC +						MSS	60,14
V13	80	90	80	90	IHC +						MSS	51,45
V14	90	95	70	80	IHC +						MSS	54,20
V15	90	95	90	90	IHC +						MSS	58,29
V16	80	80	75	80	IHC +						MSS	50,12
V17	90	95	30	90	IHC +						MSS	54,28
V18	75	40	80	85	IHC +						MSS	58,51
V19	80	85	60	80	IHC +						MSS	55,87
V20	90	90	90	70	IHC +						*MSI-H*	68,33
V21	95	95	90	90	IHC +						MSS	51,54
V22	85	90	90	85	IHC +						MSS	59,23
V23	80	90	60	75	IHC +						MSS	41,15
V24	75	80	70	75	IHC +						MSS	61,76
V25	90	95	75	70	IHC +						MSS	60,49
V26	85	90	40	75	IHC +						MSS	60,87
V27	95	90	70	85	IHC +						MSS	54,94
V28	60	80	25	55	IHC +						MSS	39,29
V29	85	90	90	80	IHC +						MSS	39,4
V30	80	95	90	80	IHC +						*MSI-H*	73,23
V31	5	90	90	0	MSI-H	is	is	is	is	is	MSI-H	80,91
V32	0	90	90	0	MSI-H	is	is	is	is	is	MSI-H	70,99
V33	1	85	70	1	MSI-H	is	is	is	is	is	MSI-H	81,24
V34	2	90	90	1	MSI-H	is	is	is	is	is	MSI-H	72,12
V35	95	98	85	2	MSI-H	is	is	is	is	is	MSI-H	65,67
V36	1	90	80	1	MSI-H	is	is	s	is	is	MSI-H	75,18
V37	1	70	60	1	MSI-H	is	is	is	is	is	MSI-H	67,41
V38	95	1	1	90	MSI-H	is	is	s	is	is	MSI-H	65,45
V39	1	90	80	1	MSI-H	is	is	is	is	is	MSI-H	79,00
V40	1	90	90	5	MSI-H	is	is	NA	is	NA	MSI-H	96,94
V41	1	60	80	5	MSI-H	is	is	is	is	is	MSI-H	66,94
V42	1	90	65	5	MSI-H	is	is	is	is	s	MSI-H	81,71
V43	2	60	50	1	MSI-H	is	is	is	s	s	MSI-H	76,46
V44	1	85	70	0	MSI-H	is	is	ND	ND	ND	MSI-H	87,92
V45	2	90	95	1	MSI-H	is	is	is	is	is	MSI-H	76,30
V46	0	95	90	0	MSI-H	is	is	is	is	is	MSI-H	88,93
V47	1	90	85	1	MSI-H	is	is	ND	ND	ND	MSI-H	89,47
V48	10	90	70	10	MSI-H	is	is	is	is	s	MSI-H	73,45
V49	1	90	80	1	MSI-H	is	is	is	is	is	MSI-H	86,46
V50	1	95	65	1	MSI-H	is	is	is	is	is	MSI-H	81,49
V51	1	45	20	1	MSI-H	is	is	is	is	is	MSI-H	66,95
V52	1	70	50	1	MSI-H	is	is	is	is	is	MSI-H	84,33
V53	1	98	80	2	MSI-H	is	is	is	is	is	MSI-H	82,68
V54	1	85	70	1	MSI-H	is	is	is	is	is	MSI-H	69,04
V55	1	90	70	1	MSI-H	is	is	is	is	is	MSI-H	81,61
V56	1	90	80	1	MSI-H	is	is	is	is	is	MSI-H	73,24
V57	1	80	25	1	MSI-H	is	is	is	is	is	MSI-H	71,72
V58	5	95	95	2	MSI-H	s	is	is	is	s	MSI-H	63,54
V59	2	90	85	5	MSI-H	is	is	is	is	s	MSI-H	64,60
V60	0	90	75	1	MSI-H	is	is	is	is	is	MSI-H	71,96

Two of the 30 MSS patients were misclassified. (Abbreviations: FLA – fragment length analysis, IHC + - no missing MMR proteins, is – instable, s – stable, NA – DNA quality insufficient, ND – not done following Bethesda guidelines, when BAT25 + BAT26 instable).

## Results

### Label-free digital pathology

**Figure 1:**
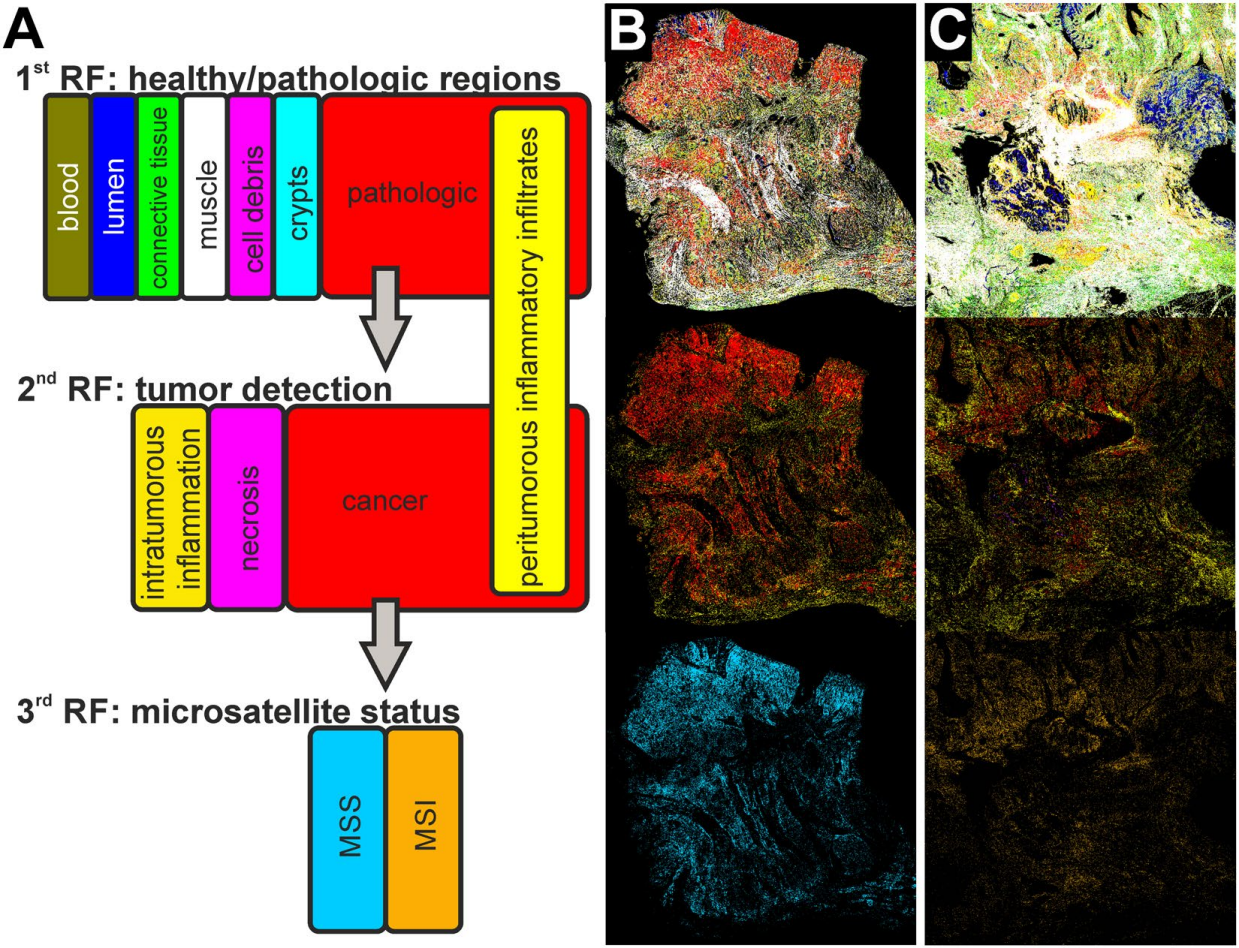
Analytical results of label-free digital pathology for colorectal cancer (CRC) diagnostics. In (**A**), the random forest (RF) classifier structure and the corresponding color code are shown schematically. The first RF (first row) determines healthy and pathologic regions, the second RF (second row) further classifies the pathologic regions to identify cancer regions, and the third RF (third row) determines microsatellite status of cancer regions. In (**B**) and (**C**), the resulting infrared (IR) index color images are shown for a microsatellite stable (MSS) CRC (**B**) and high microsatellite instable (MSI-H) CRC (**C**).

In the label-free digital pathology approach utilized here, thin tissue sections were measured by a QCL-based IR microscope. All analyzed samples are from patients with sporadic CRC UICC Stage II/III without previous chemotherapy (for details please refer to the material and methods section and Supplementary Tables 1 and 2). Spatial-resolved IR spectra reflect the integral biochemical composition of tissue like a personalized fingerprint. These fingerprints are then assigned to tissue morphological and molecular classes based on an established bioinformatics workflow using machine-learning algorithms. Here, we trained random forest (RF) classifiers to translate spectral information to a color image index representing tissue classification using a computer-stained image similar to an annotated H&E-stained one used in histopathology. All patient samples in this feasibility study were surgical samples that were measured by whole tissue section imaging of resection-sized tissue sections (up to 40 × 15 mm). Tissue microarray samples were not used. Tissue images are shown as magnified cut-outs of representative regions. The classification workflow is schematically shown in Fig. 1A. The first and second RF were presented previously with 96% sensitivity and 100% specificity^44^. In the present feasibility study we wanted to demonstrate a subsequent RF that was able to differentiate MSI-H and MSS based on the spectra recognized as tumor cells by the previously presented cancer classifier.

**Figure 2:**
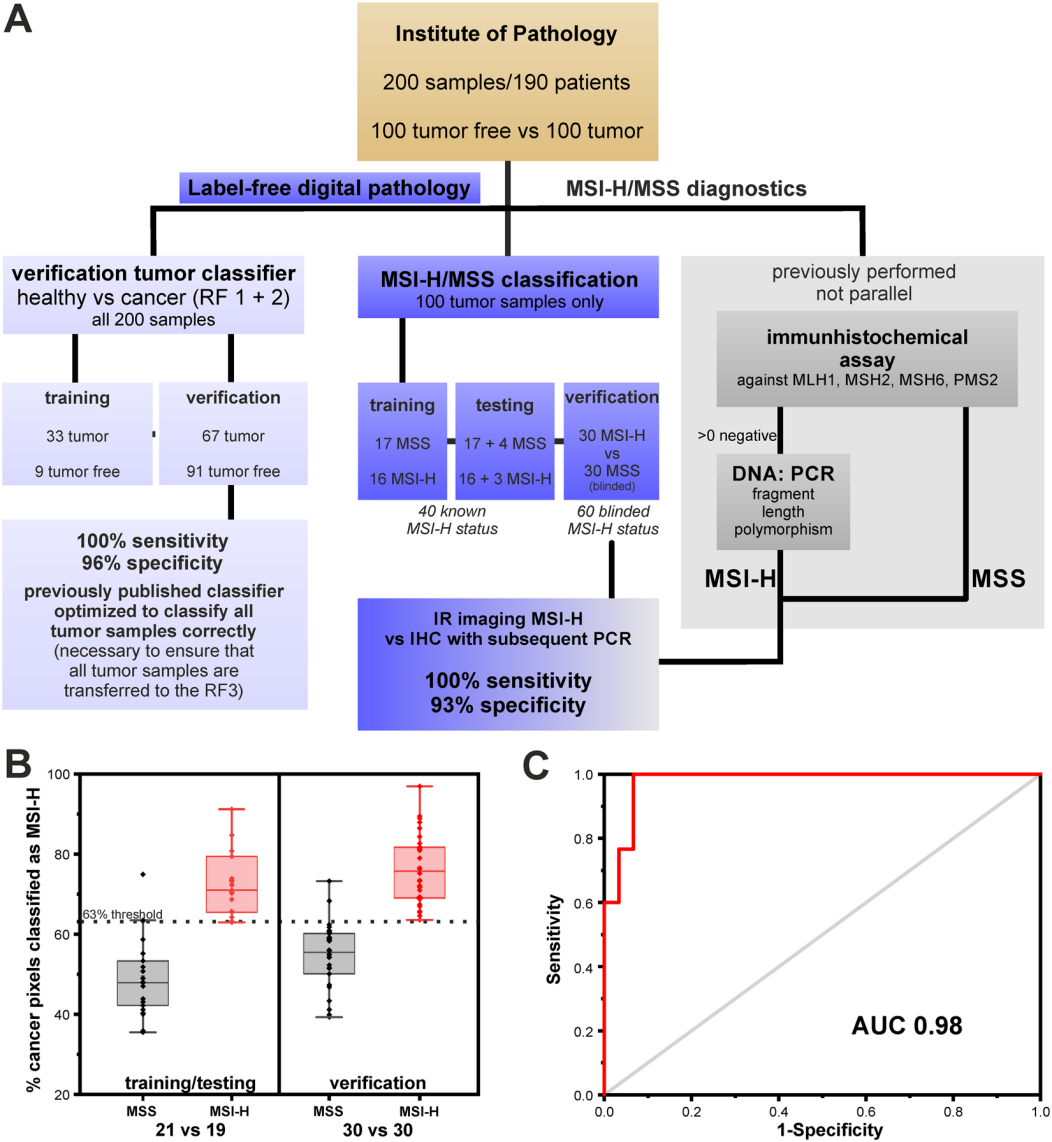
Study design, percentage results for microsatellite instability-high (MSI-H)/microsatellite stable (MSS) training/testing and verification, and receiver operating characteristic (ROC) curve of the third random forest (RF). (**A**) Study design for the presented feasibility study. Patient samples from the Institute of Pathology were previously analyzed in the clinic, including MSI-H status on adjacent thin sections. This procedure for MSI-H status is shown on the right in grey. Samples provided for label-free digital pathology are used on the one hand for optimization of the previously published cancer classifier (RF 1 + 2), shown on the left and for the feasibility study regarding MSI-H classification, shown in the middle. (**B**) Here, the percentage distribution of MSI-H spectra for the classification of MSI-H and MSS patients is shown. On the left, the results for the training/testing cohort of 40 patients is shown. The 63% threshold was set in such a way that all MSI-H tumors are reliably detected. On the right, the results for the 60 patient verification cohort are shown. The two groups can be separated by the previously determined threshold. (**C**) Curve results from varying the threshold for MSI-H positivity. The classifier reached an area under the curve (AUC) of 0.98 for the independent verification cohort of 60 patients.

Therefore, we optimized and adjusted the training of tumor classifiers to achieve a sensitivity of 100% required for presented molecular classification, else, not all cancerous samples will be transferred to the new RF. To achieve this, we double checked the first and second RF and found that some peri-tumorous inflammatory infiltrates were falsely classified as cancer cells. This appears reasonable, as the most similar class is that of peri-tumorous inflammatory infiltrates, as shown in Supplementary Fig. 2. To optimize training, we extended the previous training set from 18 samples from 11 patients to 42 samples from 33 patients (including the samples from the previous training set). All added samples were tumor samples to reflect variance in the immune cell, cancer, and necrosis class better by a higher number of spectra from different patients. For details regarding the sample/patient cohort used for training and verification, please refer to the material and methods section, Table 1, and Supplementary Tables 1 to 3. Nine tumor-free samples and 33 tumor samples were used for re-training of the first RF (healthy/pathologic) and second RF (tumor classification). Better representation of patient variations in the training dataset allowed us to decrease our tumor detection threshold from 2% to 1%. This is the sum of red pixels, denoting tumor cells in the second RF, as a ratio to all tissue pixels in the entire tissue sample, as these signals might also be obtained by misclassification due to e.g., spectral noise. The threshold is defined empirically to obtain the lowest number of false positives for tumor detection. The first RF (Fig. 1A, first row; trained on 116,117 spectra) was used to distinguish between healthy and pathologic tissue regions. The pathologic spectra are then presented to a second RF (Fig. 1A, second row; trained on 67,100 spectra) that discriminates among cancer, necrosis, and intra-tumorous inflammation (Fig. 1B,C, second row). The peri-tumorous inflammatory infiltrates (yellow) shown in the second RF are obtained by the first RF. They remained unmodified by the second RF but were found to achieve a better morphological representation of the tumor microenvironment in the resulting IR images. The ratio of inflammatory infiltrates to cancer cells appears higher for most MSI-H samples; this has been reported before and is in accordance with that observed in daily clinical routines^46,47^. Due to the study design, however, no IHC of immune cells was performed, thus, a statement here would be speculation and is therefore not discussed further. The study design is illustrated in Fig. 2A.

The re-trained combination of first and second RF reached a sensitivity of 100% and a specificity of 96% for sample-based tumor classification, with the independent cohort of 158 samples from 157 patients (true positives (TPs): 67; false negatives (FNs): 0; true negatives (TNs): 87; false positives (FPs): 4). The precise breakdown of which sample was used for which purpose is shown in Supplementary Table 3.

A sensitivity of 100% for tumor classification was essential to guarantee that all tumor samples are presented to the newly developed third RF for MSI-H detection. The third RF was trained on 33 tumor samples, which were also used for the first and second RF. Based on the results of the second RF, cancer regions (red pixels in the second RF) were further classified using a third newly developed RF (Fig. 1A–C, third row; trained on 38,261 spectra), which discriminates between microsatellite stable (MSS, light blue) and MSI-H (orange). Spectral differences between tumor cells and other cells in tissue were more evident than the minute differences between MSI-H and MSS (Supplementary Fig. 3). Due to these minuscule differences, the classification here had to be made on the second derivative of spectra. Nevertheless, only a weak classifier could be established, which is able to form a ratio from MSI-H or MSS assigned spectra. More precisely, the classifier always detects a high number of both MSI-H and MSS spectra due to small spectral differences and represents the results in a winner takes it all manner. Therefore, only one color is visible for MSS or MSI-H. In the training/testing, a threshold of 63% for MSI-H classified pixel spectra was empirically determined, which means that all tumors with more than 63% MSI-H pixel out of all tumor pixels are classified as MSI-H and represented in their respective color, MSI-H in orange and MSS in light blue. In the training/testing phase, we trained on 33 of the 40 patients whose MSI-H status was known, but tested against all 40. Therefore, the testing cohort is not independent and biased but only a few thousand spectra out of millions for each patient were used for training which could explain why it functions that well. This revealed that if a threshold of 63% of detected MSI-H spectra is applied, the two groups can be separated such that all MSI-H are correctly classified (see Fig. 2B for percentage distribution). With this threshold, we verified detection on 60 tumor samples with unknown MSI-H status (independent blinded verification set of 30 MSI-H and 30 MSS samples). In Fig. 2B, the percentage distribution for the 60 tumorous samples is shown. A good separation between the two groups can be seen. With this, a sensitivity of 100% and specificity of 93% were achieved based on comparison to sample-based diagnosis by the gold standard IHC with subsequent PCR for MSI-H (TPs: 30; FNs: 0; TNs: 28; FPs: 2). By varying the threshold, we calculated a receiver operating characteristic (ROC) curve and obtained an AUC of 0.98 (Fig. 2C). The patient-based results for gold standard methods, IHC and PCR, in comparison to whole tissue thin section IR imaging results are presented in Table 1 for the verification group of 60 patients, and in Supplementary Table 1 for the training/testing patients.

### Label-free digital pathology in comparison to histology and IHC

**Figure 3:**
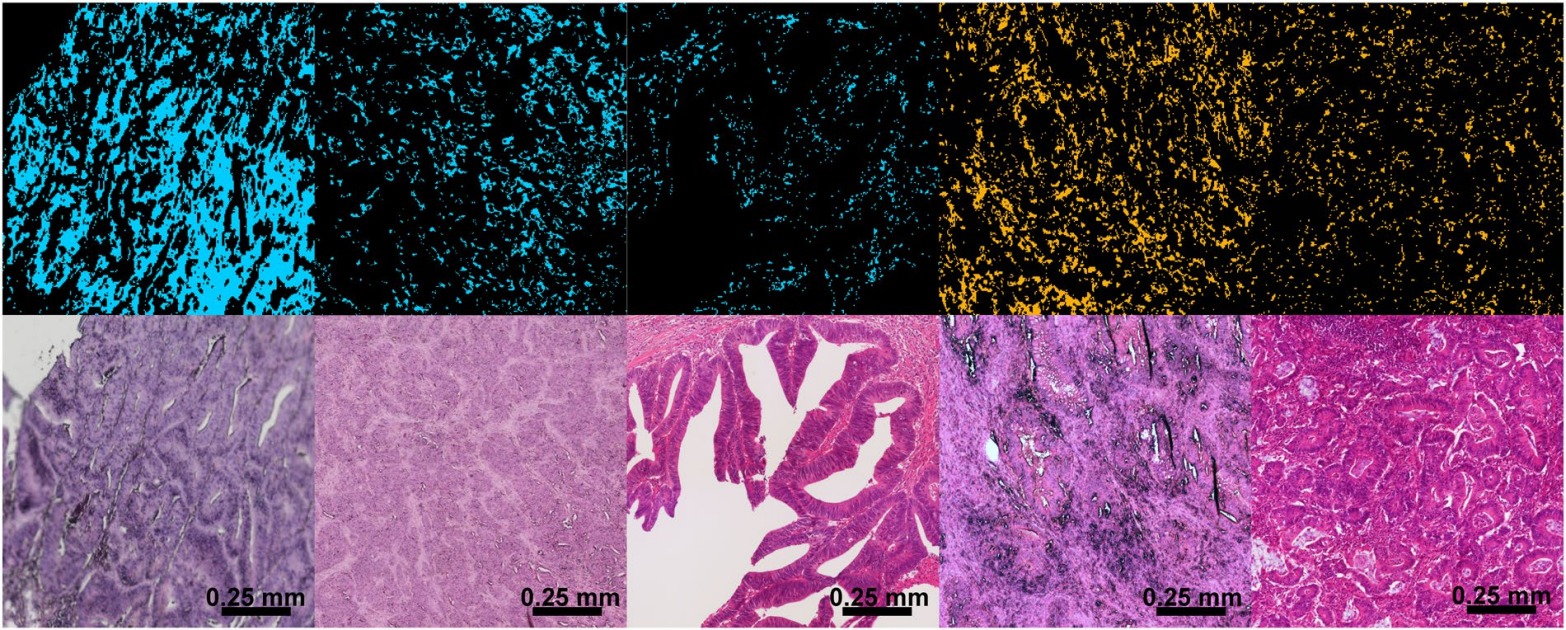
Infrared (IR) imaging for microsatellite stable (MSS; light blue) and high microsatellite instability (MSI-H) versus hematoxylin and eosin (H&E) staining of the same tissue section. Shown are example images for three MSS colorectal cancers (CRCs; light blue) and two MSI-H CRCs (orange). The IR images present cancer spectra only; therefore, the background with other tissue types is black.

To confirm the classification results for differential diagnosis of MSI-H, we compared our findings with that of histological staining and IHC. In Fig. 3, five representative CRC tissue regions are shown. The first three are MSS and the remaining two are MSI-H. The label-free MSI-H/MSS classification of the third RF is shown in the upper row (light blue: MSS, orange: MSI-H). Only pixels classified as cancer in the second RF are shown, whereas all others are black. The respective H&E staining can be seen in the lower row. From this, the morphological diversity can be clearly seen. In literature, it was found that MSI-H status is associated with a poor differentiation and high infiltration of lymphocytes in the tumor microenvironment^48–52^. It appears that the majority of MSS samples (the first three tissue regions) demonstrated a better degree of differentiation than MSI-H tumors. A detailed analysis of the dedifferentiation is not in the scope of this feasibility study and was therefore not discussed. Overall, label-free classifications (MSI-H in orange, MSS in light blue) were in accordance with the overall cancer diagnosis and were confirmed by consensus of two clinical pathologists for these small regions. The MSI-H status is difficult to determine from the H&E image.

**Figure 4:**
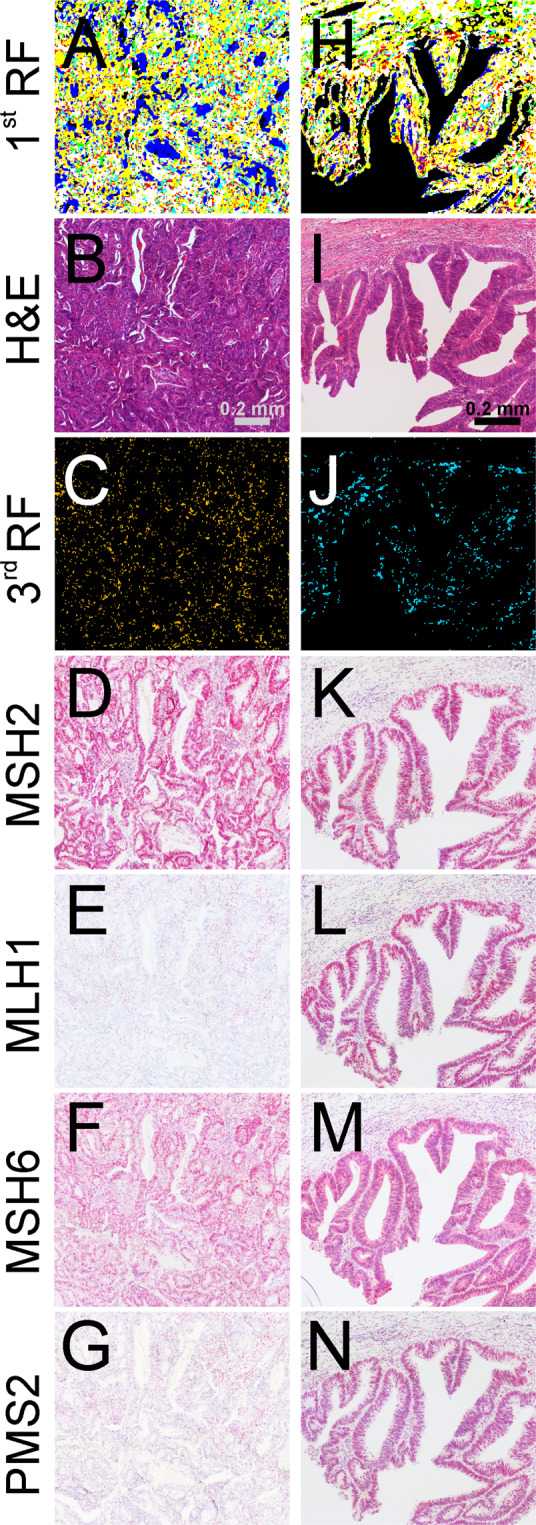
Infrared (IR) imaging for a microsatellite instability-high (MSI-H) and stable (MSS) colorectal cancer (CRC) sample (light blue) compared to hematoxylin and eosin (H&E) staining and immunohistochemistry (IHC). IR imaging results of exemplary CRC tissue regions (**A**,**C**,**H**,**J**) are shown in comparison to H&E staining (**B**,**I**). Shown in (**A**,**H**) are muscle (white), infiltrating inflammatory cells (yellow), connective tissue (green), and pathologic regions (red) and in (**C**,**J**), microsatellite status is shown as follows: MSI-H in orange (**C**) and MSS in light blue (**J**). For more details on RFs, please refer to Fig. 1A. In D-G and K-N, the four IHC protein markers MSH2, MLH1, MSH6, and PMS2 are shown for adjacent tissue sections. For MSI-H, negative staining for MLH1 (**E**) and PMS2 (**G**) indicates the defect in these mismatch repair (MMR) proteins.

Therefore, each tumor was analyzed for nuclear expression of MLH1, PMS2, MSH2, and MSH6 by IHC on adjacent tissue thin sections, and expression was noted as a percentage of positive tumor cells. Cases with an expression ratio of >10% for all four genes were classified as MSS. A comparison of the label-free digital pathology approach with IHC is shown in Fig. 4 for MSI-H (A-G) and MSS (H-N). The color image index of the first RF (A, H) and the third RF (C, J) is shown, compared to H&E staining (B, E), and the four IHC reactions (D, K: MSH2, E, L: MLH1, F, M: MSH6, and G, N: PMS2). The morphology of high-grade MSI-H tumors was often diffuse, and showed higher infiltration of immune cells, as described in other studies (please also refer to Supplementary Fig. 1)^47,53^. The MSI-H example shown (A-G) illustrates that MLH1 and PMS2 were not expressed in this tumor (E, G). For MSS (H-N), all MMR genes were expressed (K-N). A comparison of label-free classification and the usual method using IHC with subsequent PCR showed congruent results for the overall patient-based diagnosis. The more diffuse representation of the tumor in the label-free approach, in relation to H&E and IHC, resulted from the necessity to precisely classify the cancer pixel spectra in the second RF and the lower spatial resolution of IR imaging compared to brightfield microscopy. During the training phase of the classifier system we learned that the MSI-H recognition is extremely sensitive to these variances in spectral signals between these groups. The reason for this could be that the spatial resolution and, therefore, the field of view of a single detector pixel, is relatively large to resolve cell compartments. Heterogeneity of tumor along with its microenvironment may play an important role in this matter as well. Several tumors investigated in this study are highly infiltrated by immune cells, and in sum, show less distinct cancer cells. Therefore, in Fig. 4 it appears that fewer cells are IR stained compared to the strong purple staining in the H&E image. Thus, at first glance it appears inconsistent with H&E or IHC, but should be seen in a more molecular point of view. In our previous studies, we tried to represent the tumor as more compact following morphology, but for the microsatellite status, it does not result in good classifications. Therefore, we decided to represent the tumor in the manner in which we did it here to obtain the best result for tumor and microsatellite status. These differences are furthermore based on the fact that the label-free digital pathology approach was not able to differentiate which MMR gene is defective. The resulting spectral signal is based on changes in molecular composition of cell occurrence due to defective MMR genes. Thus, IHC and label-free digital pathology do not represent the same. Therefore, an accurate pixel-based comparison between the two techniques can only be made by noting that the resulting signal is in the same regions, but cannot be superimposed on exact pixel positions.

## Discussion

In previous studies, we and other groups have shown that FTIR-based IR imaging can classify tumorous tissue reflecting the annotation of pathologists, based on morphological and molecular changes in histological images. The current feasibility study showed that the faster but more complicated (owing to coherence effects) QCL-based IR imaging can also classify disease specific molecular changes. Furthermore, here, it can detect even the subtle changes due to a defective DNA mismatch repair system that cannot be reliably diagnosed by a pathologist based on H&E staining alone. Compared to the state-of-the-art IHC testing method with subsequent fragment length analysis for MMR deficiency, it is automated, label-free, requires less sample material (only one tissue thin section is needed), and is not dependent on antigen retrieval, a primary antibody, and a staining platform.

Owing to the current study design, we selected retrospective formalin-fixed, paraffine-embedded (FFPE) samples from sporadic UICC stage II/III patients, but this approach could also be applied to fresh frozen tissue by re-training RF classifiers or switching to deep learning algorithms. Recent progress in applying deep learning algorithms to hyperspectral data allows transfer learning from FFPE samples to fresh frozen ones for the same entity^54^. Following a standard dewaxing procedure for FFPE samples, no further processing of the thin tissue sections is needed. The stability of the approach for different fixation protocols is directly dependent on the dewaxing protocol used. If the protocol is well conducted, fixation influence is minimized as shown previously. Furthermore, studies on tissue microarrays from several places of origin have also shown that IR imaging is less dependent on fixation protocols. Unfortunately, the use of samples from a single clinic in this study did not allow us to draw conclusions regarding this issue from the results presented. A larger multicenter validation study is thus needed.

In the current label-free digital pathology approach, morphological features of healthy and pathologic regions including the tumor environment, cancer cells, and tumor infiltrating immune cells, as well as molecular features of microsatellite status, can all be classified in one image. This substitution of one image for all the other steps of standard diagnosis by IHC means that the whole analysis takes only approximately 30 minutes for a whole tissue thin section, including the tumor microenvironment. Therefore, we implemented the data analysis in a parallel manner to the measurement. Once the first raw data tile (480 × 480 pixels with 2 × 2 mm field of view) is written to the hard disk, the algorithm starts the preprocessing and analysis. The final RF index color image illustrating the first tile is ready before the second tile is written to the hard disk. In this manner, the diagnostic image is consecutively built during data acquisition and is finalized moments after the measurement. Further speed-up could be obtained by measuring only discrete frequencies, as reported for example, by Bhargava *et al*. and Gardner *et al*. Unfortunately, this did not allow correction for scattering effects, due to the very tiny spectral differences in our final classification MSI-H/MSS, we decided to measure whole spectra.

For MSI-H and MSS, based on the sample diagnosis level, the presented approach achieves 100% sensitivity and 93% specificity compared to those with IHC with subsequent fragment length analysis for MSI-H, resulting in a high AUC of 0.98. All patients with>63% of cancer spectra are classified as MSI-H on a sample basis. This is due to the aforementioned small differences between the classes, which lead to a weak classifier for single spectra. However, for a higher number of spectra, the classifier in combination with the threshold leads to stable results as shown in this feasibility study. The threshold was selected based on the training/test data to achieve a sensitivity of 100%. This would allow the same sample to be subsequently tested for suspected MSI-H using fragment length analysis or NGS, which are the most reliable detection techniques. In contrast to IHC, the presented approach is not able to differentiate which MMR gene is defective because it classifies MMR deficiency on an integral molecular signal not specific for single proteins. Therefore, a spatial resolved comparison of IR imaging and IHC has limitations. Thereafter, statistics were performed on a patient-based sample diagnosis level. All 30 MSI-H patient samples were correctly classified, whereas only two of the 30 MSS patient samples were falsely classified. Referring to IHC and histology results of falsely classified samples, no suspicious characteristics were found. Due to the current study design, this could not be further investigated. Only samples that showed the loss of expression of one or more MMR proteins in IHC were analyzed subsequently with fragment length analysis. Therefore, whether the falsely classified MSS specimens showed abnormal characteristics such as being MSI-like, could not be clarified. Furthermore, the large majority of analyzed patients showed MMR deficiency for MLH1 and PMS2 (Table 1). Only V35 with PMS2 and V38 with MSH2 and MSH6 have other MMR deficiencies. Therefore, it is not possible to generalize that all MMR deficient tumors will be correctly classified with the presented approach. The conclusion seems valid for MLH1/PMS2 only. Nevertheless, the two patients with different deficiencies are also correctly classified. To verify the possibility of generalization, a study with a larger cohort and FLA for all patients in a multicenter approach must be conducted to further verify and validate the results. All samples in the presented study were sporadic CRC; therefore, it could be difficult to identify Lynch syndrome patients with the presented method, due to the aforementioned limitations to resolve specific defective MMR genes. The suitability for Lynch syndrome patients must also be tested in future. However, the performance of this assay at the current stage appears comparable to that of MMR IHC. However, further studies must first illustrate to what extent IR imaging can supplement or perhaps even replace the IHC, in particular the MMR IHC, someday.

Another question that we could not address owing to the study design is whether the IR imaging can be used to analyze biopsies for MMR deficiency. In our opinion, the spectral approach here is limited at the moment. This is because, as opposed to the morphological inspection by a pathologist, the tumor microenvironment is not considered in the final classification. For example, tumor infiltrating immune cells, which are handled in literature as an important indication for the characterization of MMR deficient tumors, are currently not further classified. Therefore, we plan to extend the representation of the tumor microenvironment for specific classification of immune cells in future studies. Furthermore, the use of deep learning approaches could overcome this shortcoming but here, larger collectives are also needed to verify this.

In summary, the current study proved that QCL-based IR imaging can not only rapidly classify tissue based on morphology but also provide a more in-depth classification by resolving even subtle molecular differences due to defective DNA mismatch repair between tumor cells. This study showed that the presented label-free MSI-H CRC classification scheme based on IR imaging could become a valuable method to simplify diagnostic decisions. It requires less sample material and classifies the entire tissue and microsatellite status in approximately 30 minutes for a normal surgical sample. Furthermore, the unaltered sample material is still available for subsequent analysis with omics-based methods using IR-guided LCM, as shown in previous studies for lung cancer and bladder cancer^25,55^. Following, IR imaging could become a diagnostic tool that is quite easy to handle, as has been demonstrated in this feasibility study for stage II/III of CRC. In future, the classification could be more precise by including morphological observations in addition to spectral classification. This could be done using deep learning approaches comparable to their use in the digital pathology field. A parallel determination of the tumor environment and microsatellite status could also help to select patients for immunotherapy. Further larger multicenter translational studies are needed to validate its applicability and the benefit of deep learning approaches, for which the presented study hopefully provides a further incentive in the medical field.

## Material and Methods

### Ethical statement

All methods and experimental protocols were approved by the relevant institutional review boards (registration number 4453-12, Ethics Commission, Faculty of Medicine, Ruhr-University Bochum). Furthermore, all enrolled patients provided informed consent that the samples may be used for retrospective analysis. All procedures were conducted in accordance with the approved guidelines and regulations for human experimental research.

### Sample cohort

We obtained retrospectively 100 sporadic CRC samples, 49 MSI-H and 51 MSS, from 100 patients from the Institute of Pathology, Ruhr University Bochum, Germany. The samples were selected from a cohort including patients with primary UICC-Stage II and III tumors older than 18 years without undergoing prior chemotherapy by our pathologists. Details on tumor patients can be found in Tables 1, 2 and Supplementary Tables 1, 2. The samples are equally distributed to UICC stage II and III, none were metastatic, the majority is T3. The youngest patient was 39 years old and the oldest was 97 years old resulting in a median age of 78.

**Table 2:** Statistical data for tumor patient cohort.

	N	MSI-H	MSS
**N**	100 (100%)	49 (49%)	51 (51%)
***UICC stage***			
II	59 (59%)	33 (33%)	26 (26%)
III	41 (41%)	16 (16%)	25 (25%)
***T category***			
T1	0 (0%)	0 (0%)	0 (0%)
T2	4 (4%)	1 (1%)	3 (3%)
T3	81 (81%)	42 (42%)	39 (39%)
T4	15 (15%)	6 (6%)	9 (9%)
***N category***			
N0	59 (59%)	32 (32%)	27 (27%)
N1	21 (21%)	7 (7%)	14 (14%)
N2	19 (19%)	9 (9%)	10 (10%)
NX	1 (1%)	1 (1%)	0 (0%)
***M category***			
M0	100 (100%)	49 (49%)	51 (51%)
*sex*			
male	39 (39%)	10 (10%)	29 (29%)
female	61 (61%)	39 (39%)	22 (22%)
***localization***			
Cecum (C18.0)	20 (20%)	13 (13%)	7 (7%)
acending colon (C18.2)	26 (26%)	18 (18%)	8 (8%)
right colonic flexure (C18.3)	7 (7%)	3 (3%)	4 (4%)
transverse colon(C18.4)	11 (11%)	7 (7%)	4 (4%)
left colonic flexure (C18.5)	7 (7%)	2 (2%)	5 (5%)
descending colon (C18.6)	6 (6%)	2 (2%)	4 (4%)
sigmoidic colon (C18.7)	23 (23%)	4 (4%)	19 (19%)
Median Age	78	79	74

Tumor-free samples were obtained retrospectively from 100 patients. From 10 patients, both tumor-free and tumor samples were obtained. In summary, 200 samples from 190 patients were analyzed for optimization and evaluation of the previously published classifier for cancer recognition. For all samples, the tumor status was known. For the current feasibility study, 100 tumor samples were examined in detail. The microsatellite status for 40 tumor samples was known. Sixty tumor samples were used as a blinded verification set for MSI-H/MSS classification. The complete feasibility study design is illustrated in Fig. 2A. Tumor and tumor-free samples were two groups of the same size. For MSI-H and MSS, 50 samples for each were planned. Unfortunately, it comes out that 51 samples were MSS. This case, with the incorrect MSI-H status, was in the test cohort. The cohort size did not reflect the epidemiological distribution.

### Sample preparation

Samples were collected during surgery. Following fixation, they were handled according to standardized protocols used at the Institute of Pathology. FFPE colorectal cancer tissue blocks were cut into 7-µm thick sections and floated onto Leica frame slides with a 1.4-µm thick PET membrane. Tissue was dewaxed using established standards in groups of 10 prior to spectral data acquisition. Each slide was loaded with one sample. No tissue microarrays were used, as was mostly indicated in former IR imaging studies, and all analyzed tissues were clinical routine samples from surgeries.

### Spectral data acquisition

All measurements were performed using two Spero QT (Daylight Solutions, CA, USA) QCL-based microscopes as previously described in further detail^44^. Data acquisition was performed using chemical vision software (Daylight Solutions, CA, USA). We used the installed 4× 0.3 NA objective lens for large scale measurements covering a 2 × 2 mm^2^ field of view. The mounted uncooled microbolometer focal plane array detector was 480 × 480 pixels large, resulting in a pixel size of 4.25 × 4.25 µm. Full spectra were obtained in the range of 1800–948 cm^−1^ with a spectral resolution of 2 cm^−1^ using the transmission mode.

### Spectral processing and analysis

Spectral maps were pre-processed using the previously described workflow^30^. Strong spectral artifacts that might emerge from folds or cracks in tissue were eliminated by quality control based on the signal-to-noise ratio and the integral of the Amid I band. The remaining spectra were subjected to a Mie correction based on EMSC in the wavenumber range of 1800 to 950 cm^−1^^56^. The supervised RF classification for tissue differentiation and cancer classification was based on unsmoothed Mie corrected spectra as previously described^30,44^. For differential diagnosis of MSI-H and MSS, the RF classification was based on smoothed (9-point Savitzky-Golay) second derivative spectra. For both methods, analysis was performed on the fingerprint region from 1750 to 1000 cm^−1^.

### Classifier set-up and spectral database generation

In this study, we used the workflow established and described in our previous publications^30,31,44^. The RF classifier was shown to be a robust and reliable classifier for biomedical imaging^30–33^, and three consecutive RF analyses were used. The original training of the first two RF analyses is described elsewhere and reached 96% sensitivity and 100% specificity^44^. Here, we verified and optimized the results of this RF to reach 100% sensitivity. This was necessary to guarantee that all cancer patients can be analyzed by the third RF which was based on RF 1 + 2. Therefore, the number of tissue samples in the training and testing was raised from 18 to 42. Moreover, the results for RF 1 + 2 were verified against an independent verification set with 158 tissue samples. Please refer to Supplementary Table 3 for more details on sample sets. Spectra classified as CRC by the second RF were subjected to the third RF analysis. During the training stage for the third RF, a spectral database was generated and tested based on the 19 known MSI-H and 21 MSS CRC samples, and the MSI-H and MSS CRC samples were identified based on the representative spectra. For validation of the third RF, the blinded verification set was used (30 vs 30 samples). The RF was built with 500 trees using 16 spectral features randomly selected per decision in the trees. The most significant features for the RF were in Amid I, Amid II, and between 1300 to 1000 cm^−1^. All computations were performed in MATLAB (Mathworks, Natick, MA, USA). The final tissue annotation was provided as index color images and compared to that of corresponding H&E- and IHC-stained tissue images. For statistical analysis, IR imaging sample-based diagnoses were compared to clinical diagnoses based on IHC with subsequent PCR fragment length analysis for MSI-H. Pathologists at the Institute of Pathology, Ruhr University Bochum, provided their section analyses as well.

### Diagnostics for MSI-H/MSS using CRC tissue thin sections

Immunohistochemical analysis was performed as described previously^57^. The material was routinely fixed in 4% formaldehyde solution and embedded in paraffin. Following slicing into 4-µm-thick sections, preparations were dewaxed in xylene and subsequently rehydrated. Endogenous peroxidase activity was blocked with 3% hydrogen peroxide in methanol for 30 minutes. Following a short rinse with phosphate buffered saline, sections were pre-incubated with avidin-biotin (Camon SP 2001) for 15 minutes to reduce non-specific background staining. Preparations were covered with normal goat serum for 20 minutes and subsequently incubated with primary antibodies as follows MLH1: BD, Becton Dickinson (51–1327GR), 1:100; PMS2: Zytomed (MSK064-05), 1:100; MSH2: BD, Becton Dickinson (556349), 1:300; MSH6: BD, Becton Dickinson (610919), 1:100. Thereafter, sections were washed with phosphate-buffered saline, incubated with biotinylated goat anti-mouse immunoglobulin G (BioGenex, Germany) for 30 minutes, and covered with peroxidase-conjugated streptavidin (Dako, Denmark). Antibody detection was carried out with the Bond Polymer Refine Red Detection system as per the manufacturer’s instructions (DS9390; Leica Microsystems). Slides were counterstained with hematoxylin. Negative controls were also achieved by replacing the primary antibody with mouse or goat ascites fluid (Sigma-Aldrich Biochemicals, St. Louis, MO).

Thereafter, slides were examined and scored. Each tumor was assessed for nuclear expression of MLH1, PMS2, MSH2, and MSH6, and expression was noted as a percentage of positive tumor cells. Cases with an expression of more than 10% for all four genes were classified as MSS.

For fragment length analysis, genomic DNA was extracted from neoplastic and corresponding non-neoplastic, microdissected paraffin tissue. Fragment length changes were determined for mononucleotide markers BAT25 and BAT26, as well as dinucleotide markers D2S123, D5S346, and D17S250, by multiplex PCR in combination with high-resolution capillary electrophoresis. Cases with instability in two or more genes were subsequently classified as MSI-high, whereas cases with instability in a single gene were classified as MSI-low, according to current Bethesda criteria.

## Supplementary information

Supplementary material.

## Data Availability

The data that support the findings of this study are available from the corresponding author KG upon reasonable request.
