# Supplementary material for: Label-free, automated classification of microsatellite status in colorectal cancer by infrared imaging
Source: Sci Rep. 2020 Jun 23;10:10161. doi: 10.1038/s41598-020-67052-z (PMC7311536; doi:10.1038/s41598-020-67052-z)
Supplement: Supplementary file 1 — Supplementary material. [file 41598_2020_67052_MOESM1_ESM.pdf]

Supplementary material

Label-free, automated classification of microsatellite status in colorectal cancer by infrared imaging

Authors: Angela Kallenbach-Thieltges[1,2†], Frederik Großerueschkamp[1,2†], Hendrik Jütte[3†], Claus Kuepper[1,2], Anke Reinacher-Schick[4], Andrea Tannapfel[3], Klaus Gerwert[1,2*]

Affiliations:

[1] Ruhr University Bochum, Center for Protein Diagnostics (ProDi), Biospectroscopy, Germany.

[2] Ruhr University Bochum, Faculty of Biology and Biotechnology, Department of Biophysics, Germany.

[3] Institute of Pathology, Ruhr University Bochum, Germany.

[4] Department of Hematology, Oncology and Palliative Care, St. Josef Hospital, Ruhr University Bochum, Germany.

* To whom correspondence should be addressed: gerwert@bph.rub.de

† These authors contributed equally.

Supplementary Figure 01: H&E stained tissue thin section of a high grade MSI-H CRC sample in comparison to the corresponding diagnostic IR image.

Supplementary Figure 02: Mean spectra of the training data for selected classes with standard deviation.

Supplementary Figure 03: Mean spectra of the training data for MSI-H and MSS tumor cells.

Supplementary Table 01: IHC, PCR and IR imaging results for the 40 training/testing patients

Supplementary Table 02: Available metadata for all tumor patients

Supplementary Table 03: Breakdown of patient samples according to their use in the presented feasibility study.

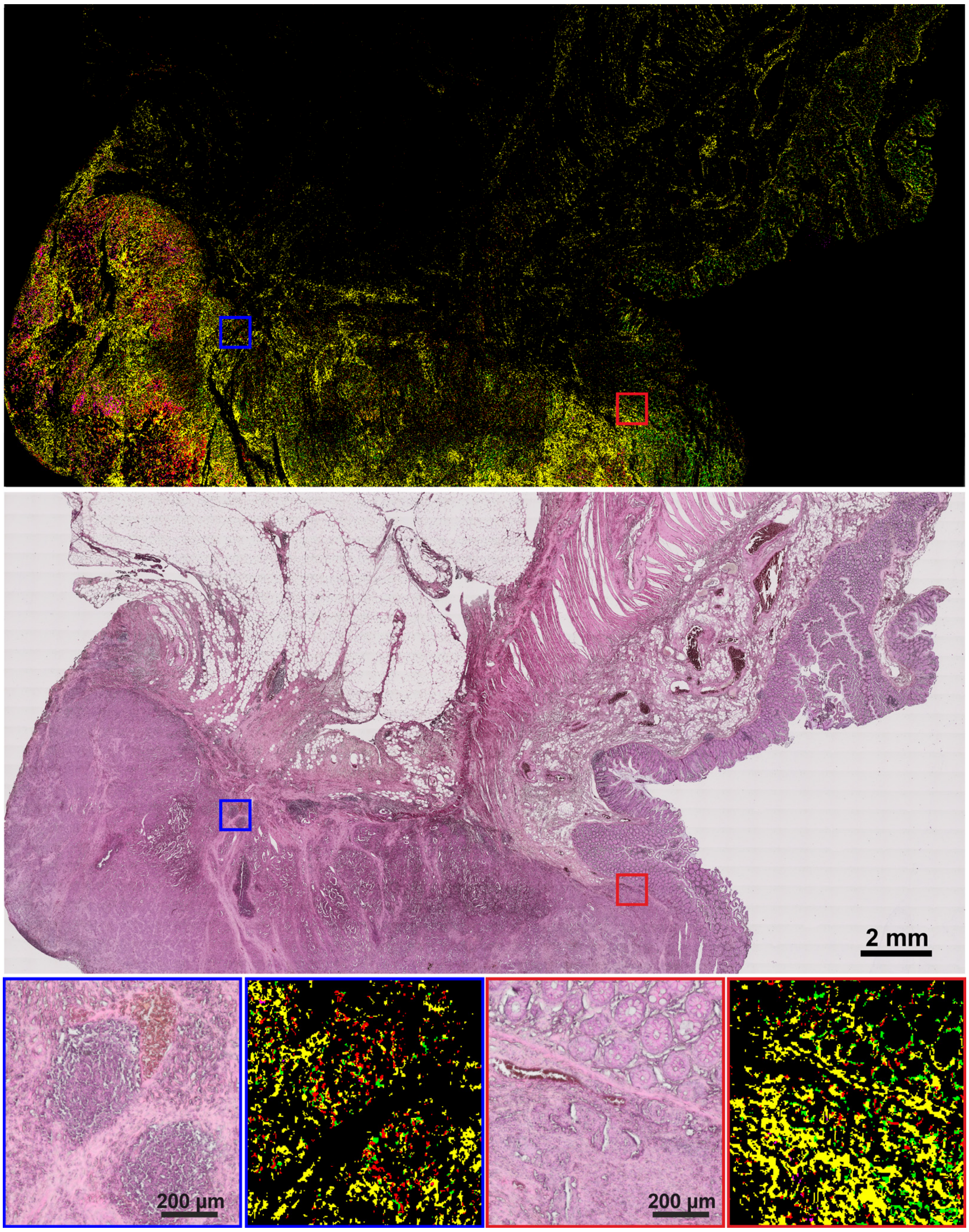

Supplementary Figure 01: H&E stained tissue thin section of a high grade MSI-H CRC sample in comparison to the corresponding diagnostic IR image. The IR image resulting of the second RF shows inflammatory infiltrates in yellow and tumor regions in red. Necrosis is marked in magenta. It is visible in the magnification cut outs that the tumor (blue rectangle) and its microenvironment (red rectangle) are massively infiltrated by immune cells.

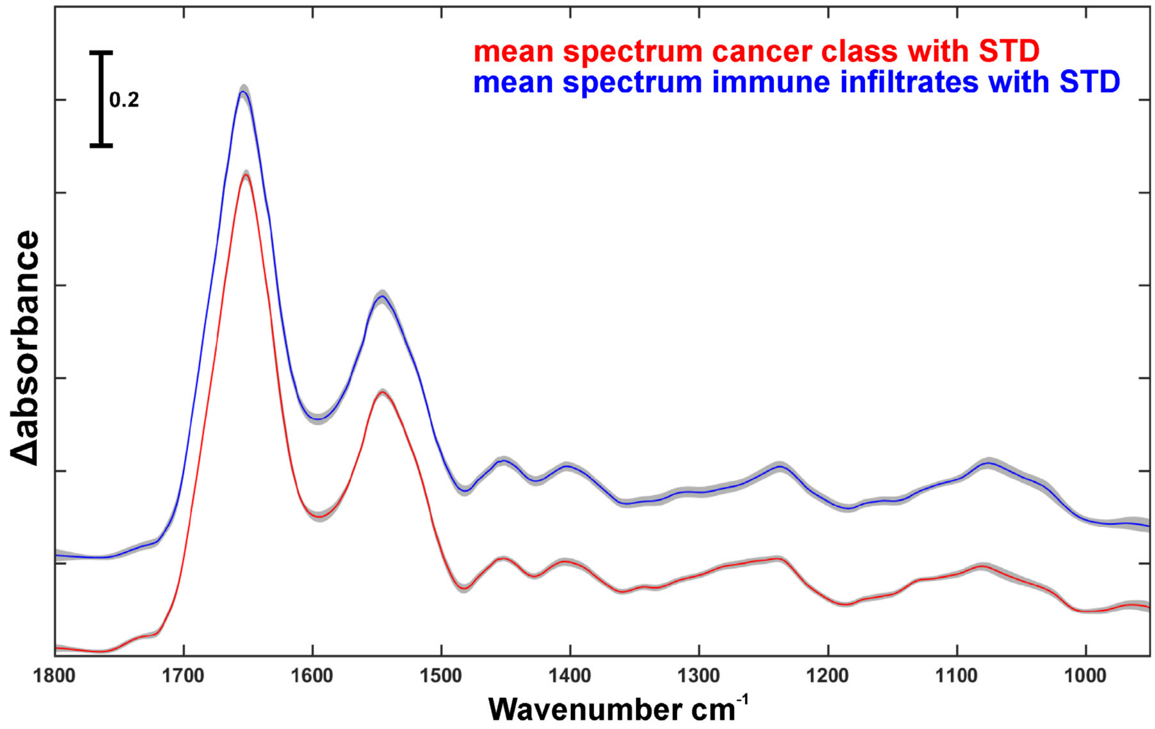

Supplementary Figure 02: Mean spectra of the training data for selected classes with standard deviation. Red spectrum, the mean of the cancer class from the second RF. Blue spectrum, the mean of immune infiltrates training spectra. The grey area marks the standard deviation over the training data. It can be seen that spectral differences between cancer and peri-tumorous immune infiltrates are quite small. Well defined classes with thousands of spectra are needed to represent these subtle differences.[30]

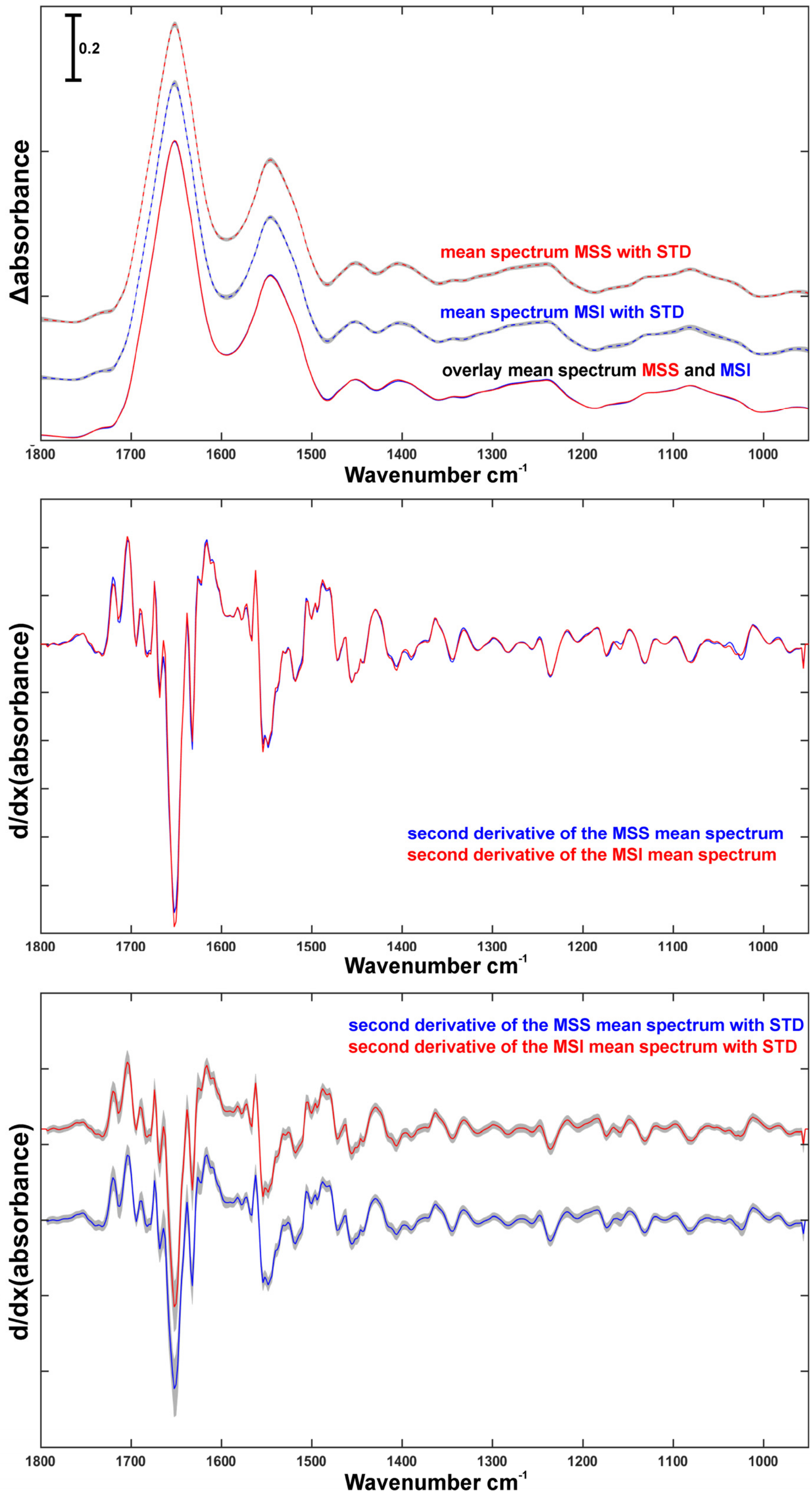

Supplementary Figure 03: Mean spectra of the training data for MSI-H and MSS tumor cells. In all panel MSI-H in blue and MSS in red. The dashed and solid lines are representing

the same. The grey area marks the standard deviation over the training data. For better visualization, the spectra and their second derivatives are shown as overlay without STD and shifted with STD. There are only very small differences between the spectral classes. Therefore, the training and preparation of training data for the third RF classifier is much more complicated than for other spectral class.

Supplementary Table 01: The table shows the IHC, PCR and IR imaging results for the 40 training/testing patients. Two of the 21 MSS patients were misclassified. (Abbreviations: FLA – fragment length analysis, IHC+ - no missing MMR proteins, is – instable, s – stable, NA – DNA quality insufficient, ND – not done following Bethesda guidelines, when BAT25+BAT26 instable). Patients in training/testing Tr01 – Tr33 and for testing only Te01 – Te07.

Patient	MLH1 %	MSH2 %	MSH6 %	PMS2 %	MSI-FLA	BAT25	BAT26	D17S250	D2S123	D5S346	IR imaging	IR MSI-H positive %
Tr01	2	95	70	2	MSI-H	is	is	is	is	is	MSI-H	64,28
Tr02	5	95	60	2	MSI-H	is	is	is	is	s	MSI-H	79,45
Tr03	1	90	60	1	MSI-H	is	is	is	is	is	MSI-H	73,92
Tr04	<1	75	75	<1	MSI-H	is	is	is	is	is	MSI-H	65,72
Tr05	0	95	80	0	MSI-H	is	is	s	is	is	MSI-H	65,50
Tr06	0	80	20	1	MSI-H	is	is	is	is	is	MSI-H	71,03
Tr07	0	80	70	1	MSI-H	is	is	is	is	s	MSI-H	63,28
Tr08	0	90	90	0	MSI-H	is	is	is	is	is	MSI-H	80,80
Tr09	N.A.	30	10	0	MSI-H	is	is	is	is	is	MSI-H	70,63
Tr10	0	80	70	0	MSI-H	is	is	is	is	is	MSI-H	73,52
Tr11	1	85	65	1	MSI-H	is	is	s	s	s	MSI-H	63,02
Tr12	0	85	80	0	MSI-H	is	is	s	s	s	MSI-H	72,30
Tr13	1	90	30	1	MSI-H	is	is	is	is	is	MSI-H	70,27
Tr14	0	85	70	0	MSI-H	is	is	is	is	is	MSI-H	84,74
Tr15	80	5	<10	70	MSI-H	is	is	s	is	is	MSI-H	73,28
Tr16	<1	80	30	<1	MSI-H	is	is	is	is	is	MSI-H	68,70
Tr17	80	5	<10	70	MSI-H	is	is	is	is	is	MSI-H	91,22
Tr18	60	80	80	45	IHC+						MSS	36,59
Tr19	90	95	80	75	IHC+						MSS	47,95
Tr20	80	70	60	65	IHC+						MSS	43,89
Tr21	90	95	85	85	IHC+						MSS	40,10
Tr22	45	75	70	40	IHC+						MSS	41,19
Tr23	90	95	70	85	IHC+						MSS	51,89
Tr24	80	90	75	70	IHC+						MSS	53,37
Tr25	85	80	70	75	IHC+						MSS	40,37
Tr26	80	90	75	75	IHC+						MSS	43,13
Tr27	70	80	65	75	IHC+						MSS	47,06

Patient	MLH1 %	MSH2 %	MSH6 %	PMS2 %	MSI-FLA	BAT25	BAT26	D17S250	D2S123	D5S346	IR imaging	IR MSI-H positive %
Tr29	85	90	75	90	IHC+						MSS	59,95
Tr30	85	95	80	80	IHC+						MSS	50,97
Tr31	98	95	95	80	IHC+						MSS	58,70
Tr32	100	95	95	90	IHC+						MSS	55,23
Tr33	80	85	40	70	IHC+						MSS	49,02
Te01	1	90	25	<1	MSI-H	is	is	is	is	is	MSI-H	84,76
Te02	1	90	75	0	MSI-H	is	is	s	is	is	MSI-H	63,17
Te03	80	60	25	75	IHC+						MSS	63,32
Te04	85	25	70	60	IHC+						MSS	47,94
Te05	75	90	90	50	IHC+						MSS	35,94
Te06	60	65	75	45	IHC+						MSS	48,09
Te07	65	75	90	70	IHC+						MSS	74,95

Supplementary Table 02: Available metadata for all patients. Shown are all tumor samples for the independent verification cohort (V01 – V60) and the training/testing cohort (Tr01 – Tr33 and Te01 – Te07).

Patient	UICC	T category	N category	M category	Gender	AGE
V01	2	T3b	N0	M0	f	84
V02	3	T3c	N0	M0	f	81
V03	2	T3	N0	M0	m	84
V04	2	T3b	N0	M0	m	81
V05	3	T3b	N1a	M0	m	87
V06	2	T3c	N0	M0	f	74
V07	2	T3b	N0	M0	f	68
V08	3	T3a	N1a	M0	m	70
V09	2	T4b	N0	M0	f	74
V10	2	T3c	N0	M0	f	88
V11	2	T3a	N0	M0	f	69
V12	3	T3b	N2a	M0	f	41
V13	2	T3b	N0	M0	f	53
V14	3	T3b	N2b	M0	f	80
V15	2	T3b	N0	M0	m	62
V16	2	T2b	N0	M0	f	79
V17	3	T3c	N2a	M0	m	65
V18	2	T3b	N0	M0	f	58

Patient	UICC	T category	N category	M category	Gender	AGE
V19	2	T4b	N0	M0	f	87
V20	3	T3b	N2	M0	f	82
V21	3	T3c	N1b	M0	f	71
V22	3	T3b	N2b	M0	m	80
V23	2	T3	N0	M0	m	87
V24	2	T2b	N0	M0	f	80
V25	3	T3	N1	M0	m	40
V26	2	T4	N0	M0	m	69
V27	3	T2	N1	M0	m	70
V28	3	T3	N1	M0	m	86
V29	3	T3b	N1	M0	m	80
V30	3	T3	N1	M0	m	77
V31	2	T3c	N0	M0	f	63
V32	3	T3c	N2a	M0	f	78
V33	3	T3c	N1a	M0	f	82
V34	2	T3a	N2a	M0	m	85
V35	2	T3a	N0	M0	m	63
V36	2	T3	N0	M0	f	88
V37	2	T3	N0	M0	f	78
V38	2	T3	N0	M0	m	79
V39	2	T3	N0	M0	f	77
V40	2	T3	N0	M0	f	65
V41	2	T3	N0	M0	f	59
V42	2	T3	N0	M0	f	82
V43	2	T3	N0	M0	m	80
V44	2	T3	N0	M0	m	79
V45	3	T4b	N2b	M0	f	44
V46	2	T3	N0	M0	f	87
V47	2	T3	N0	M0	f	74
V48	2	T3	N0	M0	f	68
V49	2	T3	N0	M0	f	81
V50	2	T3	N0	M0	m	70
V51	2	T3	N0	M0	m	81
V52	3	T3a	N2a	M0	f	74
V53	2	T4b	N0	M0	f	89
V54	2	T3a	N0	M0	f	76
V55	2	T3d	N0	M0	f	94
V56	2	T3b	N0	M0	m	82
V57	2	T3d	N0	M0	f	92
V58	3	T3b	N1b	M0	f	83
V59	2	T3d	N0	M0	f	78
V60	3	T4b	N1b	M0	f	79
Tr01	3	T4b	N2b	M0	m	87

Patient	UICC	T category	N category	M category	Gender	AGE
Tr02	2	T3c	N0	M0	m	39
Tr03	2	T3b	N0	M0	f	68
Tr04	2	T3b	N0	M0	f	85
Tr05	3	T2	N1b	M0	f	96
Tr06	2	T3	N0	M0	f	90
Tr07	3	T3	N1	M0	f	83
Tr08	2	T3a	N0	M0	f	79
Tr09	2	T3c	NX	M0	f	81
Tr10	3	T3c	N0	M0	f	88
Tr11	3	T3b	N2b	M0	f	90
Tr12	2	T3	N0	M0	f	89
Tr13	3	T3	N2	M0	f	76
Tr14	3	T3	N2	M0	f	91
Tr15	3	T4b	N1a	M0	f	77
Tr16	2	T3b	N0	M0	f	76
Tr17	2	T3c	N0	M0	f	79
Tr18	3	T4b	N2b	M0	f	90
Tr19	3	T3b	N1b	M0	f	77
Tr20	3	T4b	N1b	M0	f	42
Tr21	2	T3b	N0	M0	m	65
Tr22	2	T3a	N0	M0	m	66
Tr23	3	T4b	N1	M0	f	71
Tr24	3	T3b	N0	M0	m	77
Tr25	2	T3b	N0	M0	m	78
Tr26	2	T3b	N0	M0	m	75
Tr27	2	T3	N0	M0	m	69
Tr28	3	T4b	N0	M0	f	68
Tr29	2	T3b	N0	M0	m	81
Tr30	2	T3c	N0	M0	f	53
Tr31	3	T3c	N2a	M0	m	66
Tr32	3	T3b	N2a	M0	m	73
Tr33	2	T3b	N0	M0	m	71
Te01	2	T3b	N0	M0	m	76
Te02	3	T3b	N2a	M0	m	74
Te03	3	T2	N1b	M0	f	84
Te04	3	T3b	N2a	M0	m	72
Te05	3	T4	N1	M0	m	97
Te06	3	T4	N1	M0	m	84
Te07	2	T4b	N0	M0	f	65

Supplementary Table 03: Breakdown of patient samples according to their use in the presented feasibility study. Shown are all samples for the independent verification tumor cohort (V01 – V60) and the training/testing cohort (tumor samples: Tr01 – Tr33 and Te01 – Te07; tumor-free samples H01 – H10; tumor-free and tumor samples from the same patients are written in italic style). Patients used in training/testing are marked in light blue, for testing only in light orange, and all independent verification patients are marked in light green.

**Patient**	**Train RF1**	**Train RF2**	**Train RF3**	**Verify RF1+2**	**Verify RF 3**
Tr01	x	x	x		
Tr02	x	x	x		
Tr03	x	x	x		
Tr04	x	x	x		
Tr05	x	x	x		
Tr06	x	x	x		
Tr07	x	x	x		
Tr08	x	x	x		
Tr09	x	x	x		
Tr10	x	x	x		
Tr11	x	x	x		
Tr12	x	x	x		
Tr13	x	x	x		
Tr14	x	x	x		
Tr15	x	x	x		
Tr16	x	x	x		
Tr17	x	x	x		
Tr18	x	x	x		
Tr19	x	x	x		
Tr20	x	x	x		
Tr21	x	x	x		
Tr22	x	x	x		
Tr23	x	x	x		
Tr24	x	x	x		
Tr25	x	x	x		
Tr26	x	x	x		
Tr27	x	x	x		
Tr28	x	x	x		
Tr29	x	x	x		
Tr30	x	x	x		
Tr31	x		x		
Tr32	x		x		

**Patient**	**Train RF1**	**Train RF2**	**Train RF3**	**Verify RF1+2**	**Verify RF 3**
Tr33	x		x		
Te01				x	x
Te02				x	x
Te03				x	x
Te04				x	x
Te05				x	x
Te06				x	x
Te07				x	x
H01	x				
H02	x				
H03	x				
H04	x				
H05	x				
H06	x				
H07	x				
H08	x				
H09	x				
H10				x	
H11				x	
H12				x	
H13				x	
H14				x	
H15				x	
H16				x	
H17				x	
H18				x	
H19				x	
H20				x	
H21				x	
H22				x	
H23				x	
H24				x	
H25				x	
H26				x	
H27				x	
H28				x	
H29				x	
H30				x	
H31				x	
H32				x	
H33				x	
H34				x	
H35				x	

Patient	Train RF1	Train RF2	Train RF3	Verify RF1+2	Verify RF 3
H36				x	
H37				x	
H38				x	
H39				x	
H40				x	
H41				x	
H42				x	
H43				x	
H44				x	
H45				x	
H46				x	
H47				x	
H48				x	
H49				x	
H50				x	
H51				x	
H52				x	
H53				x	
H54				x	
H55				x	
H56				x	
H57				x	
H58				x	
H59				x	
H60				x	
H61				x	
H62				x	
H63				x	
H64				x	
H65				x	
H66				x	
H67				x	
H68				x	
H69				x	
H70				x	
H71				x	
H72				x	
H73				x	
H74				x	
H75				x	
H76				x	
H77				x	
H78				x	

Patient	Train RF1	Train RF2	Train RF3	Verify RF1+2	Verify RF 3
H79				x	
H80				x	
H81				x	
H82				x	
H83				x	
H84				x	
H85				x	
H86				x	
H87				x	
H88				x	
H89				x	
H90				x	
H91				x	
H92				x	
H93				x	
H94				x	
H95				x	
H96				x	
H97				x	
H98				x	
H99				x	
H100				x	
V01				x	x
V02				x	x
V03				x	x
V04				x	x
V05				x	x
V06				x	x
V07				x	x
V08				x	x
V09				x	x
V10				x	x
V11				x	x
V12				x	x
V13				x	x
V14				x	x
V15				x	x
V16				x	x
V17				x	x
V18				x	x
V19				x	x
V20				x	x

Patient	Train RF1	Train RF2	Train RF3	Verify RF1+2	Verify RF 3
V21				x	x
V22				x	x
V23				x	x
V24				x	x
V25				x	x
V26				x	x
V27				x	x
V28				x	x
V29				x	x
V30				x	x
V31				x	x
V32				x	x
V33				x	x
V34				x	x
V35				x	x
V36				x	x
V37				x	x
V38				x	x
V39				x	x
V40				x	x
V41				x	x
V42				x	x
V43				x	x
V44				x	x
V45				x	x
V46				x	x
V47				x	x
V48				x	x
V49				x	x
V50				x	x
V51				x	x
V52				x	x
V53				x	x
V54				x	x
V55				x	x
V56				x	x
V57				x	x
V58				x	x
V59				x	x
V60				x	x
